# Proctalgia and constipation secondary to hypertrophic polyglucosan inclusion body myopathy of the internal anal sphincter: a case report

**DOI:** 10.1186/s13256-018-1856-z

**Published:** 2018-10-24

**Authors:** Ioanna G Panagiotopoulou, Richard Miller, Michael P Powar, James Y H Chan, R Justin Davies

**Affiliations:** 1 Cambridge Colorectal Unit, Addenbrooke’s Hospital, Cambridge University Hospitals NHS Foundation Trust, Hills Road, Cambridge, CB2 0QQ UK; 2 Department of Pathology, Addenbrooke’s Hospital, Cambridge University Hospitals NHS Foundation Trust, Hills Road, Cambridge, CB2 0QQ UK

**Keywords:** Proctalgia, Hereditary internal anal sphincter myopathy, Polyglucosan inclusion bodies

## Abstract

**Background:**

Hereditary polyglucosan inclusion body myopathy of the internal anal sphincter is a rare cause of proctalgia fugax and constipation. Treatment options are explored.

**Case presentation:**

A 61 year-old Caucasian woman presented with an 18-year history of severe anal pain and constipation. She had no response to medical treatment which included amitriptyline and topically administered diltiazem. Endoscopy revealed no abnormalities, whereas endoanal ultrasound showed an abnormally thick internal anal sphincter (> 5 mm) and anal manometry showed intermittent episodes of very high resting pressures in excess of 200 mmHg that resolved spontaneously after 2 minutes. She had no relief of her symptoms after receiving an injection of botulinum toxin to the internal anal sphincter. She subsequently underwent a lateral internal anal sphincterotomy which led to complete resolution of her symptoms.

**Conclusions:**

Hereditary polyglucosan inclusion body myopathy of the internal anal sphincter should be considered in the differential diagnosis of a patient presenting with severe anal pain and constipation in the absence of an anal fissure or sepsis. If medical therapy with calcium antagonists fails to provide symptom relief, lateral internal sphincterotomy should be considered rather than botulinum toxin injection.

## Background

Proctalgia fugax is characterized by sudden and transient recurrent attacks of severe anorectal pain that are usually worse at night [1]. Because commonly no anorectal pathology is identified to account for the symptoms, proctalgia fugax may be considered a functional disorder that is associated with a high prevalence of psychiatric illness [2]. However, Kamm *et al.* [3] were the first to describe hereditary hypertrophy and hypertonia of the internal anal sphincter (IAS) and they suggested that a rare autosomal-dominant inherited sphincter myopathy, which presented with intermittent severe anal pain and constipation, was responsible. The histopathological changes seen in this rare condition comprise hypertrophy, vacuolation, and the presence of ovoid acid-Schiff-positive polyglucosan inclusion bodies in the smooth muscle fibers of the IAS [4]. Since the description of this rare condition, several cases have been reported recognizing the rare syndrome of both proctalgia fugax and constipation associated with IAS hypertrophy [5] both with [6–9] or without [10, 11] polyglucosan inclusion bodies on histology.

We present a further case of proctalgia and constipation due to a hypertrophic IAS whose histology was consistent with polyglucosan inclusion body myopathy. Our patient did not respond to conservative management or botulinum toxin (Botox) injections of the IAS but symptoms resolved with lateral internal anal sphincterotomy. Although polyglucosan inclusion body myopathy of the IAS is a rare condition leading to proctalgia and constipation, it is important that the colorectal surgeon treating patients with similar symptoms and signs considers this rare diagnosis and proceeds with offering the patient appropriate surgical management as suggested both by our experience and the available current literature.

## Case presentation

A 61 year-old Caucasian woman was referred to our colorectal clinic with an 18-year history of severe intermittent anal pain and constipation. She described experiencing intermittent anal spasms lasting around 15 minutes. These episodes were worse when sitting down for longer than 45 minutes or when lying in bed. The frequency of these anal spasms was increasing with time and occurring every hour at night at the time of presentation. Her constipation symptoms constituted experiencing difficulty in defecation and a sensation of incomplete evacuation. She had no response to amitriptyline or topical diltiazem. Her past medical history was unremarkable apart from four normal vaginal deliveries. Her sister had colorectal cancer diagnosed at the age of 49 and had previously been treated for an undiagnosed anal sphincter problem. There was no other relevant history of note.

**Fig. 1:**
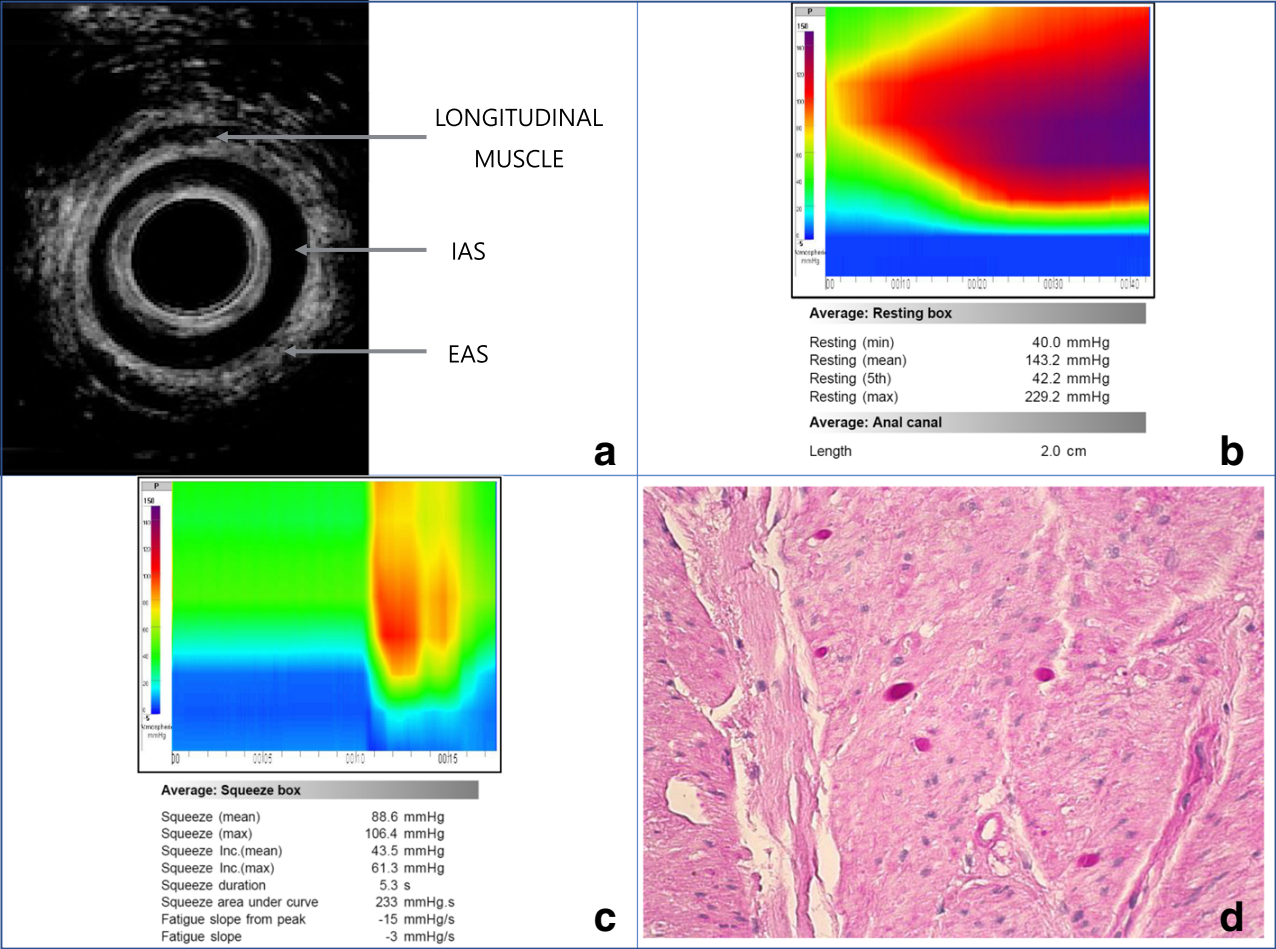
Investigations confirming internal anal sphincter myopathy. **a** Endoanal ultrasound. The internal anal sphincter is thickened with a diameter greater than 5 mm. *EAS* external anal sphincter, *IAS* internal anal sphincter. **b** Anal manometry with resting pressure exceeding 200 mmHg in internal anal sphincter myopathy. **c** Anal squeeze pressure lower than maximum resting pressures seen in anal sphincter myopathy. **d** Histology of the internal anal sphincter biopsy showing vacuolation of smooth muscle containing periodic acid–Schiff-positive polyglucosan body inclusions (hematoxylin and eosin, periodic acid–Schiff, × 20 magnification)

She initially underwent a flexible sigmoidoscopy and magnetic resonance imaging (MRI) of her perineum. The endoscopy was reported as normal, whereas the MRI showed edema of the IAS. She subsequently had an endoanal ultrasound which confirmed that her IAS was abnormally thick and greater than 5 mm (Fig. 1a). Anal manometry revealed that although resting and squeeze pressures were within normal limits there were periods of a significant increase in anal resting pressure lasting longer than 2 minutes (Fig. 1b). Pressures during this period were in excess of 200 mmHg which settled spontaneously. These pressures were even higher than the maximum recorded squeeze pressure (Fig. 1c).

She had an examination of the anal canal under anesthetic which showed a very prominent sphincter complex. She also received Botox injections (Dysport™) at the 3 and 9 o’clock positions of the IAS which led to no subsequent resolution of her symptoms. She then underwent a lateral internal anal sphincterotomy by dividing half of the length (1 cm) of the IAS on the left lateral aspect. A biopsy of the IAS taken at the time of surgery was sent for histology which confirmed polyglucosan body myopathy of the IAS (Fig. 1d). At 3-month follow-up, she had complete resolution of her symptoms and has not contacted our department with any concerns for more than 1-year postoperatively.

## Discussion

In this case report, we present a rare hereditary case of polyglucosan inclusion body myopathy of the IAS resulting in proctalgia and constipation. Our patient did not respond to conservative or medical therapy with calcium antagonists but experienced full resolution of her symptoms with lateral internal anal sphincterotomy. Our experience is in agreement with the experience of other groups [3, 6, 7, 9–11] that internal anal sphincterotomy is the treatment of choice, whereas we also report an attempt to achieve symptom relief with Botox injections that was not successful.

An isolated inherited hypertrophic myopathy of the IAS has previously been described presenting with proctalgia fugax and constipation [3–11]. The symptoms comprising this syndrome include proctalgia with its severity being worse at night or with changes in posture, whereas the term constipation is used to describe the outlet obstruction experienced secondary to a hypertrophied IAS [3–11]. Although a rare condition, its description within families supports the finding of its inheritance being autosomal dominant with incomplete penetrance [3, 5, 9]. Our patient had a family member treated elsewhere for anal sphincter symptoms and it is in all described cases of this condition that other family members are reported to experience similar symptoms even if not directly investigated [3–11].

In this rare syndrome, the hypertrophied IAS shows rare histopathological features consistent with vacuolation and the presence of ovoid polyglucosan inclusion bodies in disordered smooth muscle fibers [4]. The pathological significance of polyglucosan bodies is not clear but they have previously been found in the small and large bowel of patients with severe gastrointestinal (GI) motility disorders [12]. In contrast to other hollow visceral smooth muscle myopathies, where atrophy and multiple GI tract involvement may occur [4, 12], the myopathy of the IAS is isolated and is characterized by hypertrophy of the IAS [3–11]. Our histology results revealed polyglucosan inclusion bodies as identified in most other reported cases [3–9]. In contrast, Orkins [10] and de la Portilla *et al*. [11] did not find any polyglucosan inclusion bodies on histopathology despite the similarity of the clinical syndrome presented.

In our patient, the smooth muscle of the IAS was seen to be abnormally thick (> 5 mm) on endoanal ultrasound examination, whereas intermittent very high resting pressures were observed at anal manometry. Our finding of a thickened IAS was consistent with results from previously reported cases, where the IAS was examined with endoanal ultrasound or MRI and found to be between 5 and 12 mm thick [3–11]. Our results of anal manometry revealed normal resting and squeeze pressures with intermittent significant increases in the anal resting pressure in excess of 200 mmHg. Such high resting pressure episodes lasted longer than 2 minutes. This is in agreement with the hypertonia seen in most reported cases [3, 5, 6, 8, 10] with the maximum previously reported resting pressure being 290 mmHg [10]. Interestingly, Konig *et al.* [7] and de la Portilla *et al*. [11] found normal resting pressures on anal manometry despite identifying a thickened IAS.

The high resting pressure ultraslow waves seen in anal manometry were previously reported to coincide with episodes of proctalgia [3, 10]. Celik *et al.* [5] treated the affected patients with a sustained release formulation of the calcium antagonist nifedipine rather than any surgical intervention. The pharmacological management with nifedipine resulted in reduced anal tone and less frequent and severe attacks of anal pain [5]. In contrast to these findings, Zbar and colleagues [9] did not see an improvement in symptoms with orally administered nifedipine and they therefore proceeded with surgery. Oral calcium channel blocker therapy with diltiazem has also been reported to provide symptomatic relief in cases of refractory proctalgia following IAS strip myectomy [6, 7].

In our case, topically administered diltiazem had no effect on our patient’s proctalgia symptoms and we offered injection of Botox to the IAS. The contraction of the IAS is mediated by sympathetic innervation. Botox is used for the treatment of anal fissure [13] and is thought to stop neural transmission by irreversibly binding to the presynaptic nerve terminals and blocking the release of acetylcholine [14]. The injection of Botox had no effect on our patient’s symptoms of proctalgia or outlet obstruction. We found no other reports of Botox being used for the treatment of symptoms secondary to hereditary hypertrophic polyglucosan body myopathy of the IAS, but it was felt that it was worth an attempt in order to avoid potential risk of permanent flatus incontinence associated with any division of the IAS.

Finally, we proceeded in our patient with lateral internal anal sphincterotomy which led to resolution of her symptoms, with no disturbance to continence. Shabani *et al.* [8] did two partial internal anal sphincterotomies and reported partial relief of either proctalgia or the outlet obstruction symptoms. The surgeons involved in the rest of the case reports available to date proceeded with IAS strip myectomy [3, 6, 7, 9–11]. Strip myectomy did not alter the tone of the remaining IAS but led to a fivefold reduction in the high resting pressures [3]. The initial study by Kamm *et al.* [3] showed a cure of the outlet obstruction symptoms but partial relief of the proctalgia whose attacks became less frequent following surgery. Orkins [10] and Zbar *et al*. [9] reported rapid resolution of proctalgia symptoms following IAS strip myectomy with the expense of incontinence being experienced by the patient in the former group. The rest of the groups reported partial or no relief of proctalgia symptoms with strip myectomy [6–9] which subsequently required long-term orally administered calcium antagonist therapy [6, 9]. With regards to relief from the outlet obstruction symptoms, most report partial relief with two cases experiencing incontinence [9, 10].

## Conclusions

Our experience and the available literature on this rare inherited hypertrophic polyglucosan body IAS myopathy resulting in proctalgia fugax and outlet obstruction, show that calcium channel antagonists can be considered in the medical management of proctalgia symptoms. Botox injections do not seem to result in any symptom resolution but internal anal sphincterotomy should be considered the treatment option of choice should medical therapy fail.

## Abbreviations

Botox: Botulinum toxin
GI: Gastrointestinal
IAS: Internal anal sphincter
MRI: Magnetic resonance imaging
